# Effects of carrier solutions on the viability and efficacy of canine adipose-derived mesenchymal stem cells

**DOI:** 10.1186/s12917-021-03120-4

**Published:** 2022-01-07

**Authors:** Tania Sultana, Ahmed Abdal Dayem, Soo Bin Lee, Ssang-Goo Cho, Jeong Ik Lee

**Affiliations:** 1 Regenerative Medicine Laboratory, Center for Stem Cell Research, Department of Biomedical Science and Technology, Institute of Biomedical Science and Technology, Konkuk University, 120 Neungdong-ro, Gwangjin-gu, Seoul, Republic of Korea; 2 Department of Veterinary Obstetrics and Theriogenology, College of Veterinary Medicine, Konkuk University, Seoul, Republic of Korea; 3 Department of Stem Cell and Regenerative Biotechnology, Konkuk University, Seoul, 05029 Republic of Korea

**Keywords:** Adipose-derived mesenchymal stem cells, Canine, 5% dextrose solution, Hartmann’s solution, Heparin, Phosphate-buffered saline, Proliferation, Stemness, Transplantation, 0.9% saline

## Abstract

**Background:**

Mesenchymal stem cells (MSCs) have favorable characteristics that render them a potent therapeutic tool. We tested the characteristics of MSCs after temporal storage in various carrier solutions, such as 0.9% saline (saline), 5% dextrose solution (DS), heparin in saline, and Hartmann’s solution, all of which are approved by the U.S. Food and Drug Administration (FDA). Phosphate-buffered saline, which does not have FDA approval, was also used as a carrier solution. We aimed to examine the effects of these solutions on the viability and characteristics of MSCs to evaluate their suitability and efficacy for the storage of canine adipose-derived MSCs (cADMSCs).

**Results:**

We stored the cADMSCs in the test carrier solutions in a time-dependent manner (1, 6, and 12 h) at 4 °C, and analyzed cell confluency, viability, proliferation, self-renewability, and chondrogenic differentiation. Cell confluency was significantly higher in 5% DS and lower in phosphate-buffered saline at 12 h compared to other solutions. cADMSCs stored in saline for 12 h showed the highest viability rate. However, at 12 h, the proliferation rate of cADMSCs was significantly higher after storage in 5% DS and significantly lower after storage in saline, compared to the other solutions. cADMSCs stored in heparin in saline showed superior chondrogenic capacities at 12 h compared to other carrier solutions. The expression levels of the stemness markers, Nanog and Sox2, as well as those of the MSC surface markers, CD90 and CD105, were also affected over time.

**Conclusion:**

Our results suggest that MSCs should be stored in saline, 5% DS, heparin in saline, or Hartmann’s solution at 4 °C, all of which have FDA approval (preferable storage conditions: less than 6 h and no longer than 12 h), rather than storing them in phosphate-buffered saline to ensure high viability and efficacy.

## Background

Over the last two decades, stem cell transplantation has gained the attention of researchers due to its functional characteristics and therapeutic potential [1]. Mesenchymal stem cells (MSCs) or mesenchymal progenitor cells are recognized as the most broadly utilized stem cells in tissue engineering and regenerative medicine [2] because of their ability to treat different immune disorders [3] and their eminent multi-lineage differentiation potentiality [4]. They can be isolated from various sources, primarily from the bone marrow (BM), adipose tissue, umbilical cord blood, Wharton’s jelly, etc. [5]. Among these, AT is considered an abundant source of MSCs [6]. Furthermore, adipose-derived MSCs (ADMSCs) have shown therapeutic success in pre-clinical studies in various fields as they are more immune-privileged [7] and significantly more genetically stable for extended culture [7] compared to BM-derived MSCs (BMMSCs) [8]. Various pathologies are similar in canines and humans; canines thus portray an ideal model for replicating human conditions. They can also serve as a replacement for laboratory animals with artificially created diseases [9]. Consequently, canine ADMSCs (cADMSCs) can be considered a potential tool for stem cell-based therapy and as a model for human diseases.

Clinical applications of MSCs are determined by different factors such as the number of cells used [10], cell sources [11], culture conditions [12], cell collection processes [13], transplantation time [14], inter-individual variation [15], implementation methods [16], and cell quality before transplantation. Approval from the U.S. Food and Drug Administration (FDA) can also be a criterion for selecting carrier solutions before clinical therapy. Being fragile and extremely sensitive, MSCs require a strictly maintained environment to retain their quality and prevent deterioration. To minimize the decline in cell quality, it is advisable to transplant MSCs immediately after harvesting. However, time differences, ranging from a few hours to several days, between cell harvesting and transplantation are often unavoidable. Before clinical therapy, MSCs are stored in carrier solutions, followed by direct transplantation into the body. However, it is necessary to investigate the effects of the currently available carrier solutions on MSCs to determine which of these solutions best maintain cell viability and biological functions.

We selected five of the most commonly used carrier solutions in MSCs transplantation for clinical and experimental purposes, namely 0.9% saline (saline) [17], phosphate-buffered solution (PBS) [18], 5% dextrose solution (5% DS) [19], heparin in saline (Hepa-Sal) (1 IU/mL) [20], and Hartmann’s solution (HS) [21].

Saline is useful for maintaining cell viability and high proliferation ability [22], and 5% DS containing dextrose in saline for intravenous administration may contribute to hyperglycemia [23] and has a significantly negative effect on the cell metabolism and viability of MSCs in culture [24]. Hepa-Sal is composed of heparin, commonly used as an anticoagulant for the treatment of embolisms or thromboses [25], and saline. Heparin is also used in cell culture to boost the beneficial effects of extracellular supplements that are used; for instance, heparin does not affect the viability of human ADMSCs (hADMSCs) for at least 24 h [20], and it promotes cell proliferation [26, 27], cell viability [28], and differentiation of MSCs [29, 30]. A recent case of sudden death caused by hepatic embolism after infusion with MSCs attracted global attention to the acute toxicity of MSCs [31]. Li Liao et al. have suggested anticoagulation treatment with heparin for the efficient prevention of MSCs-induced coagulation and the adverse effect of high-dose MSCs infusion [32]. HS is an isotonic solution of several salts that are non-toxic to most cells [33], and it has more clinical benefits compared to those of physiologic saline [34]. Finally, unlike the aforementioned FDA-approved carrier solutions, PBS is applicable in laboratory protocols as additives in cell culture media [35]. Additionally, it is also used as the carrier solution of MSCs before transplantation for clinical or experimental purposes [36]. However, if PBS is used as a carrier, cell washing using FDA-approved solutions should be performed before transplantation.

In this study, we aimed to investigate the currently approved carrier solutions (saline, 5% DS, Hepa-Sal, and HS) as well as PBS in a time-dependent manner to determine the appropriateness and efficacy of these carrier solutions for the storage of ADMSCs at 4 °C.

## Results

### Effects of carrier solutions on cell morphology and proliferation

**Fig. 1:**
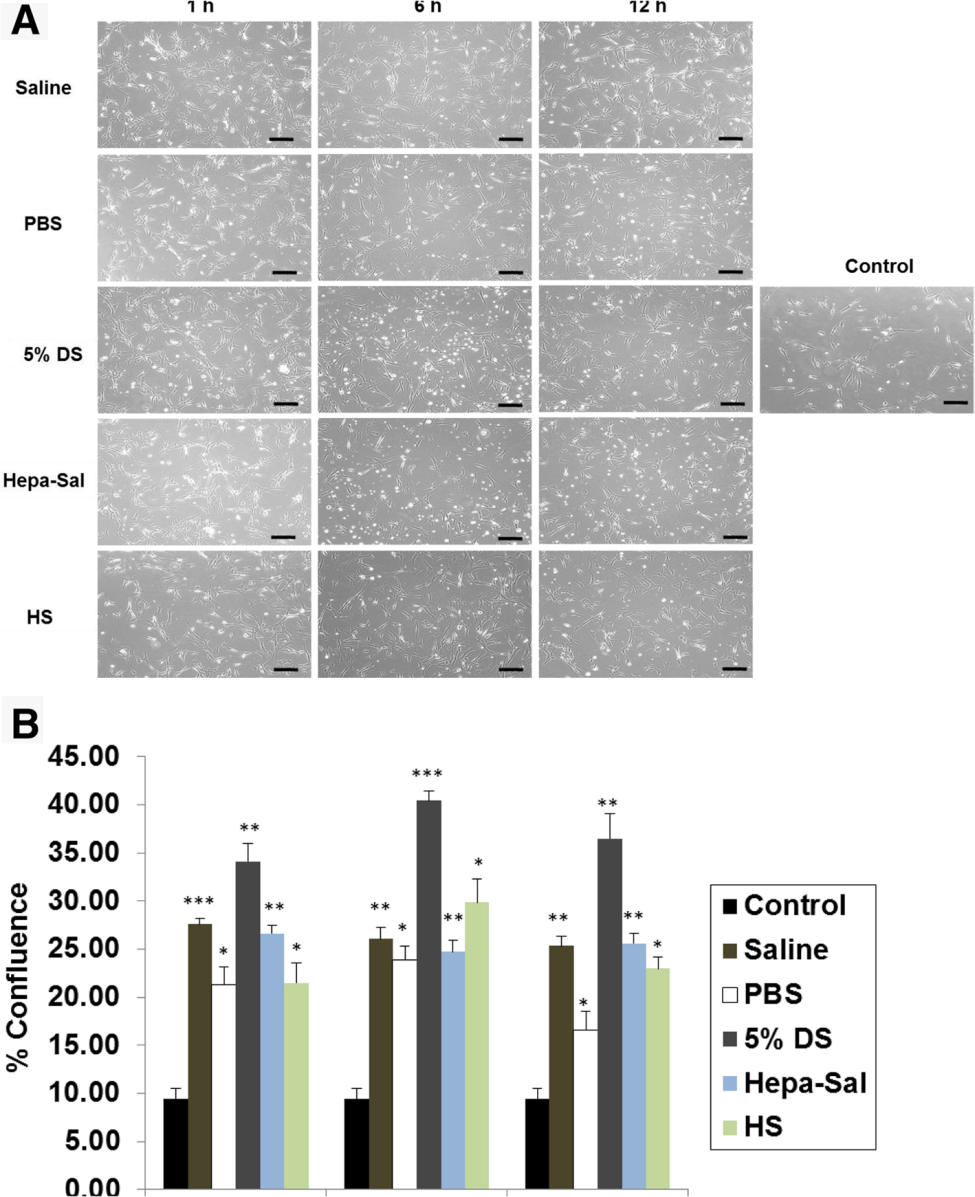
Morphology and confluency of post storage cADMSCs. **A** After storage in different carrier solutions at 4 °C in a time-dependent manner, cADMSCs at 24 h showed the typical fibroblast-like morphology (Scale bar = 50 μm). Cell confluency of cADMSCs was measured using ImageJ software. **B** cADMSCs stored in 5% DS showed the highest confluence until 12 h, whereas cells stored in PBS for 1, 6, and 12 h showed the lowest confluence at each time point compared with other solutions. Cells not stored in carrier solutions served as controls. cADMSCs - canine adipose-derived mesenchymal stem cells, Saline - 0.9% saline, PBS - Phosphate-buffered saline, 5% DS - 5% Dextrose solution, Hepa-Sal - Heparin in saline, HS - Hartmann’s solution

All the post-storage adherent cells had polymorphic and fibroblast-like morphology. They adhered to the plastic surface and expanded as a monolayer, which is characteristic of stem cells. No apparent morphological changes were observed among cADMSCs stored in the five different carrier solutions (Fig. 1A). cADMSCs significantly showed the highest and lowest confluency upon storage in 5% DS and PBS, respectively, for 12 h (Fig. 1B). cADMSCs stored for 1 h in saline, Hepa-Sal, and HS showed 27.61, 26.62, and 21.49% cell confluency, respectively. Storage in Hepa-Sal for 12 h showed 26.60% confluency, which is higher than that shown after storage in saline and HS for 12 h.

### pH measurements

We aimed to measure the pH of the carrier solutions, as a pH that is different from the physiological pH can lead to unexpected effects on cell viability. There was an initial increase in the pH of fresh solutions when cADMSCs were stored in them, but this decreased over time. All solutions maintained a pH range of 5.45–7.56 (Table 1), which is acceptable for parenteral transplantation.

**Table 1:** pH of selected solutions for cADMSCs

Time (h)	Saline	PBS	5% DS	Hepa-Sal	HS
0	5.24 ± 0.02	7.55 ± 0.03	4.16 ± 0.07	4.89 ± 0.03	6.38 ± 0.01
1	7.31 ± 0.17	7.56 ± 0	6.17 ± 0.73	6.69 ± 0.09	6.74 ± 0.02
6	6.96 ± 0.14	7.55 ± 0.01	6.15 ± 0.13	6.65 ± 0.07	6.67 ± 0.05
12	6.89 ± 0.12	7.55 ± 0.01	5.45 ± 0.86	6.62 ± 0.15	6.70 ± 0.05

cADMSCs were suspended in different carrier solutions at different time points at 4 °C. Fresh solutions without storing cells at time 0 were used as controls. The results are representative of three independent experiments. The results are presented as the mean ± SD. *cADMSCs* canine adipose-derived mesenchymal stem cells, *Saline* 0.9% saline, *PBS* Phosphate-buffered saline, *5% DS* 5% Dextrose solution, *Hepa-Sal* Heparin in saline, *HS* Hartmann’s solution, *SD* Standard deviation

### Effects of carrier solutions on cell proliferation

**Fig. 2:**
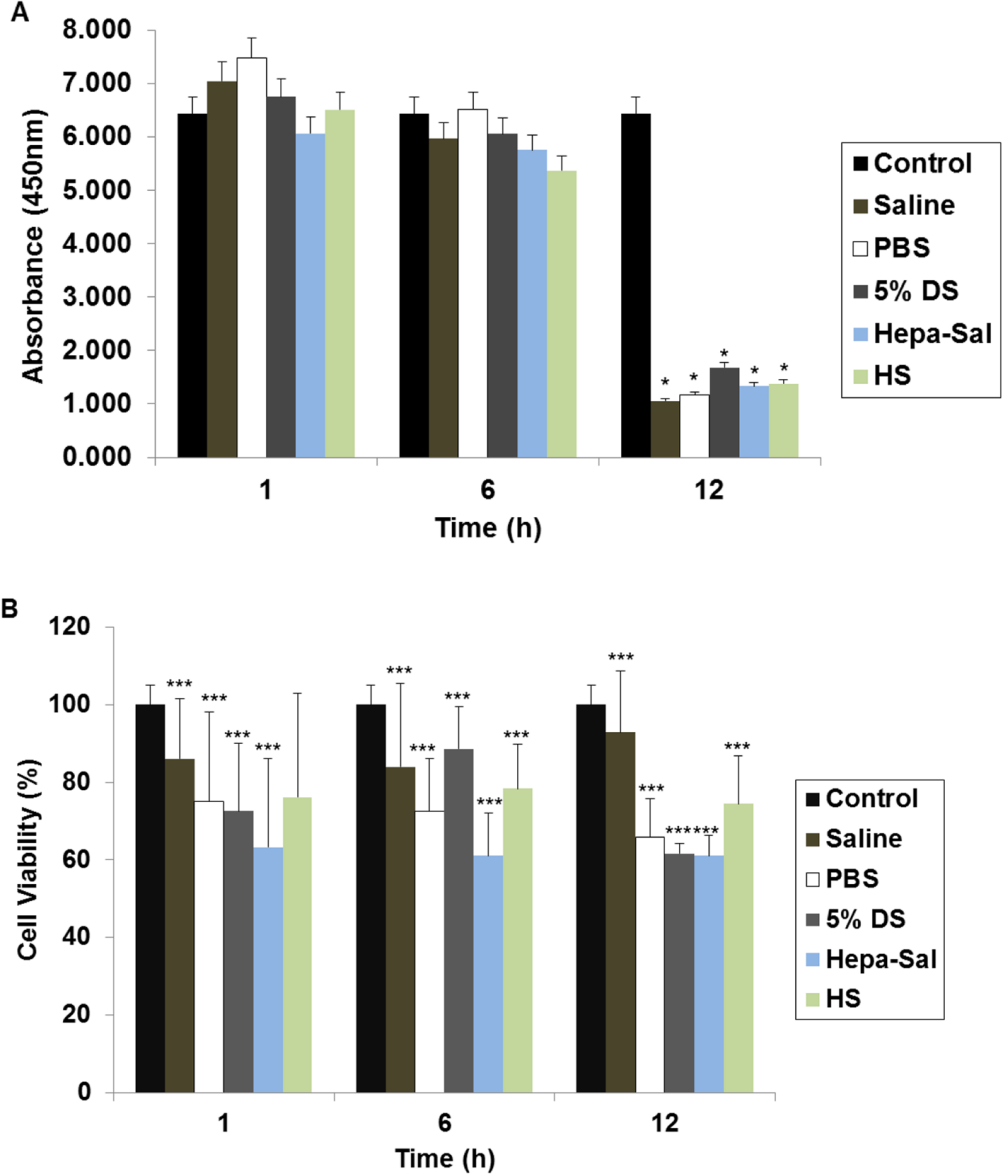
Post-storage proliferation and viability of cADMSCs. cADMSCs were suspended in different carrier solutions in a time-dependent manner at 4 °C. **A** Proliferation was determined by the cell counting kit-8 assay. cADMSCs showed no significant decrease in proliferation until 6 h of storage. However, there was significantly higher proliferation at 12 h of storage in 5% DS. **B** Viability was measured by the MTT assay. The viability of cADMSCs decreased significantly over time. Cells not stored in carrier solutions served as controls. The results are representative of three independent experiments. The bar graph represents the mean ± SD. cADMSCs - canine adipose-derived mesenchymal stem cells, Saline - 0.9% saline, MTT - 3,4,5 – dimethylthiazol-2-yl)-2–5-diphenyltetrazolium bromide, PBS - Phosphate-buffered saline, 5% DS - 5% Dextrose solution, Hepa-Sal - Heparin in saline, HS - Hartmann’s solution, SD - Standard deviation. (*P < 0.05, **P < 0.001, and ***P < 0.0001)

A high proliferation rate is a prerequisite for stem cells used in clinical applications. Cell counting kit-8 (CCK-8) assays showed that cADMSCs stored for 1 and 6 h in the five different carrier solutions showed a slight reduction or increase in proliferation, respectively (Fig. 2A). There was a significant decrease in the cell viability after proliferation at 12 h, with the highest and lowest proliferation observed in 5% DS and saline, respectively.

### Effects of carrier solutions on the viability of cADMSCs

Generally, detachment of anchorage-dependent cells from the substrate culminates in apoptosis [37]. Although MSCs are known to be less responsive to this type of apoptosis, their viability can be affected when they are detached from a fixed surface in the absence of serum components [38]. Thus, for clinical trials, the effect of carrier solutions on cell viability should be examined. After storage for 1 h, cADMSCs in saline maintained > 86% cell viability, and other carrier solutions maintained approximately 70% cell viability. The viability of cADMSCs was higher in 5% DS and saline at 6 and 12 h, respectively, and decreased significantly in a time-dependent manner in other carrier solutions (Fig. 2B).

### Effects of carrier solutions on colony-forming capacity

**Fig. 3:**
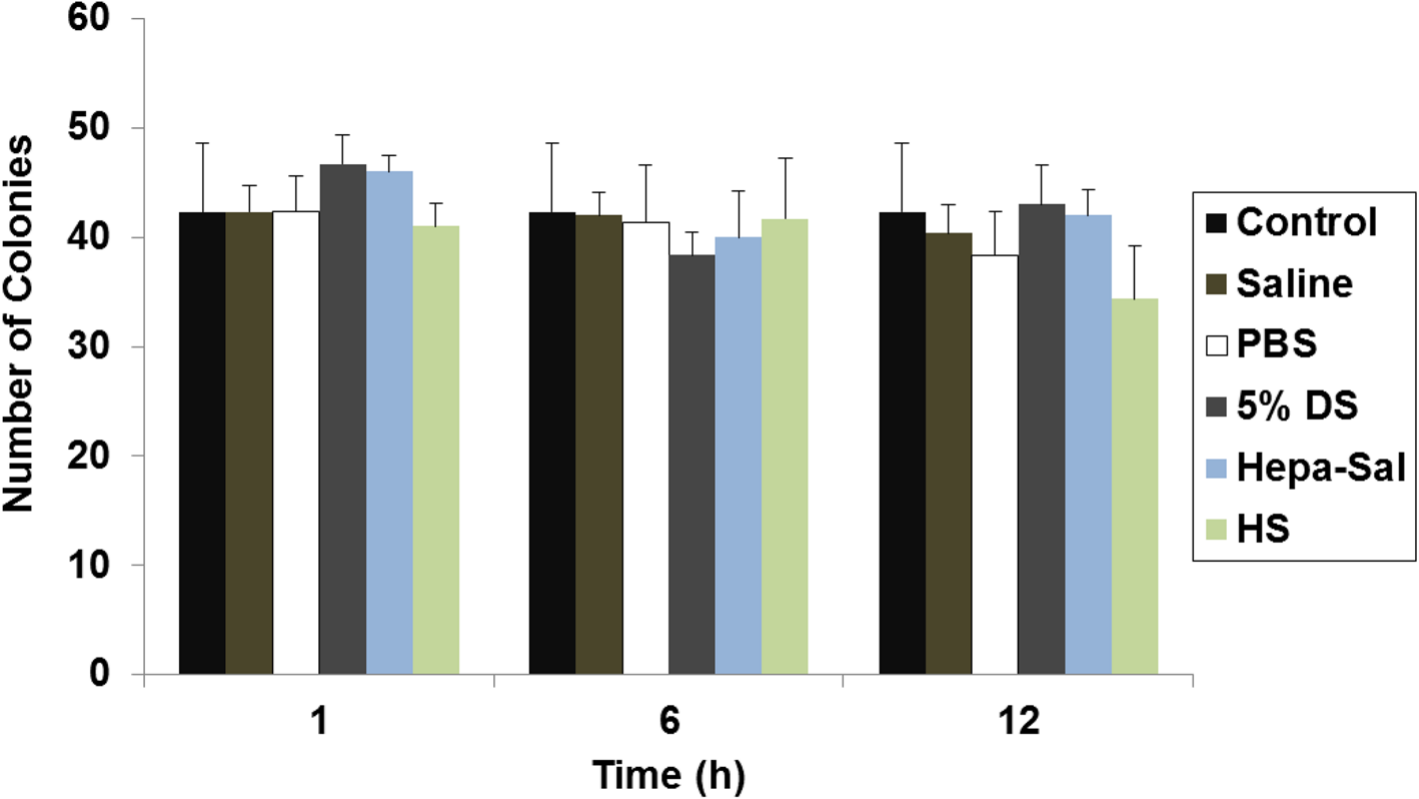
Post-storage colony-forming ability of cADMSCs. cADMSCs were suspended in various carrier solutions at 4 °C for different time points and formed colonies in all experimental groups. 1% crystal violet was used for staining and clusters with > 50 cells were considered colonies. Cells not stored in carrier solutions served as controls. The results are representative of three independent experiments. The bar graph represents the mean ± SD. cADMSCs - canine adipose-derived mesenchymal stem cells, Saline - 0.9% saline, PBS - Phosphate-buffered saline, 5% DS - 5% Dextrose solution, Hepa-Sal - Heparin in saline, HS - Hartmann’s solution

The colony-forming capacity (CFU) of cADMSCs stored in the different carrier solutions decreased over time. cADMSCs stored in saline and HS formed the highest number of colonies at 6 h (Fig. 3). After 12 h, cADMSCs in saline, 5% DS, and Hepa-Sal formed > 40 colonies, whereas approximately 34–38 colonies were formed in other solutions. There was no significant change in many colonies over time.

### Effects of carrier solutions on the expression of stemness and cell surface markers

**Fig. 4:**
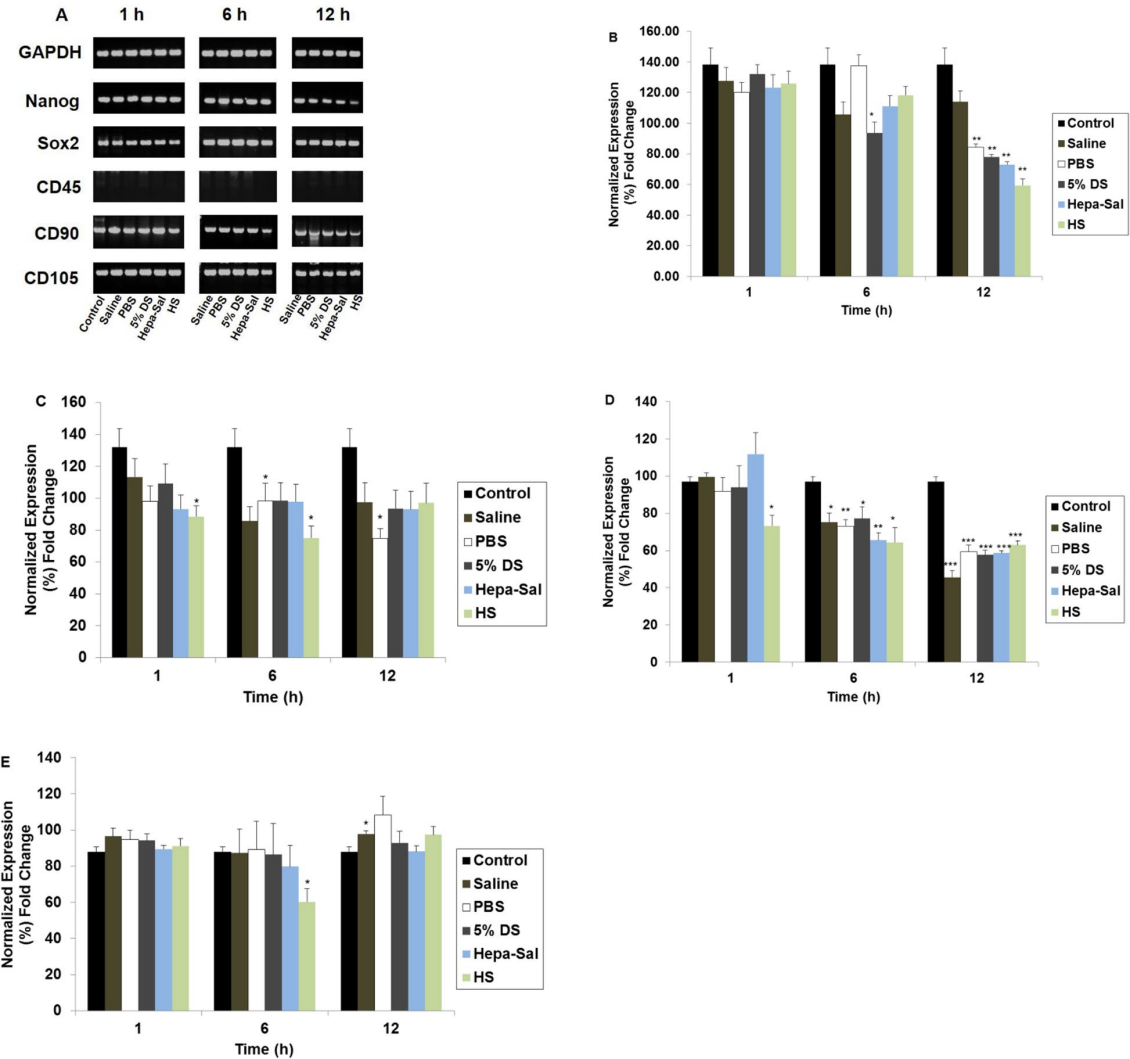
Post-storage gene expression of stemness and surface markers of cADMSCs. **A** Gel results of genes. cADMSCs were suspended in various carrier solutions at 4 °C in a time-dependent manner and analyzed by RT-PCR for the expression of Nanog, Sox2, CD45, CD90, and CD105 followed by agarose gel electrophoresis. GAPDH was used as the housekeeping control gene. cADMSCs in all solutions did not express negative CD45. **B**, **C**, **D**, **E** Post-storage quantitative analysis of gene expression levels of cADMSCs. cADMSCs were suspended in various carrier solutions at 4 °C in a time-dependent manner. GAPDH was used as the housekeeping control gene. All mRNA data were normalized to the level of GAPDH and relative fold changes in expression level were measured. **B** The expression level of Nanog was significantly higher in HS at 1 h, which gradually decreased. Saline showed a significantly higher expression of Nanog at 12 h of storage. **C** A significantly high expression of Sox2 was found in 5% DS at 1 h and 12 h of storage. **D** The expression level of CD 90 up to 6 h was significantly higher in saline and PBS. **E** The expression level of CD105 at 12 h was significantly higher in PBS. Cells not stored in carrier solutions served as controls. The results are representative of three independent experiments. The bar graph represents the mean ± SD. cADMSCs - canine adipose-derived mesenchymal stem cells, GAPDH - Glyceraldehyde-3-Phosphate Dehydrogenase, Saline - 0.9% saline, PBS - Phosphate-buffered saline, 5% DS - 5% Dextrose solution, Hepa-Sal - Heparin in saline, HS - Hartmann’s solution, RT-PCR- Reverse transcription-polymerase chain reaction. SD - Standard deviation. (*P < 0.05, **P < 0.001, and ***P < 0.0001)

cADMSCs stored in various carrier solutions at different time points were evaluated for the gene expression, indicating stemness and other stem cell characteristics. The gene expression profiles of the housekeeping gene, Nanog, Sox2, cluster of differentiation (CD) 45, CD90, and CD105 are presented in Fig. 4A. cADMSCs stored in the different carrier solutions did not express the negative surface marker CD45. However, they expressed the positive markers CD90 and CD105, as well as the stemness markers Nanog and Sox2. Their expression was affected over time when stored in different carrier solutions.

At 6 h, cADMSCs showed the highest expression of Nanog in PBS and the lowest expression in 5% DS (Fig. 4B). cADMSCs stored in PBS, 5% DS, Hepa-Sal, and HS showed a significantly low expression level of Nanog at 12 h. Even though the expression of Nanog decreased at 6 h in saline, its expression was the highest at 12 h. PBS, 5% DS, and Hepa-Sal revealed an almost similar level of expression of Sox2 at 6 h (Fig. 4C). Sox2 expression in HS decreased gradually until 6 h but we observed higher expression at 12 h. A significantly lower expression of Sox2 was found in PBS at 12 h, and an approximately similar level of expression was observed after storage in the other solutions. Hepa-Sal revealed the highest expression of CD90 at 1 h, and the expression level was significantly higher in 5% DS and saline at 6 h and HS at 12 h (Fig. 4D). The expression of CD105 was affected over time, as it was significantly lower in HS at 6 h and significantly higher in saline at 12 h. PBS showed the highest expression of CD105 at 12 h (Fig. 4E).

### Effects of carrier solutions on chondrogenic differentiation

**Fig. 5:**
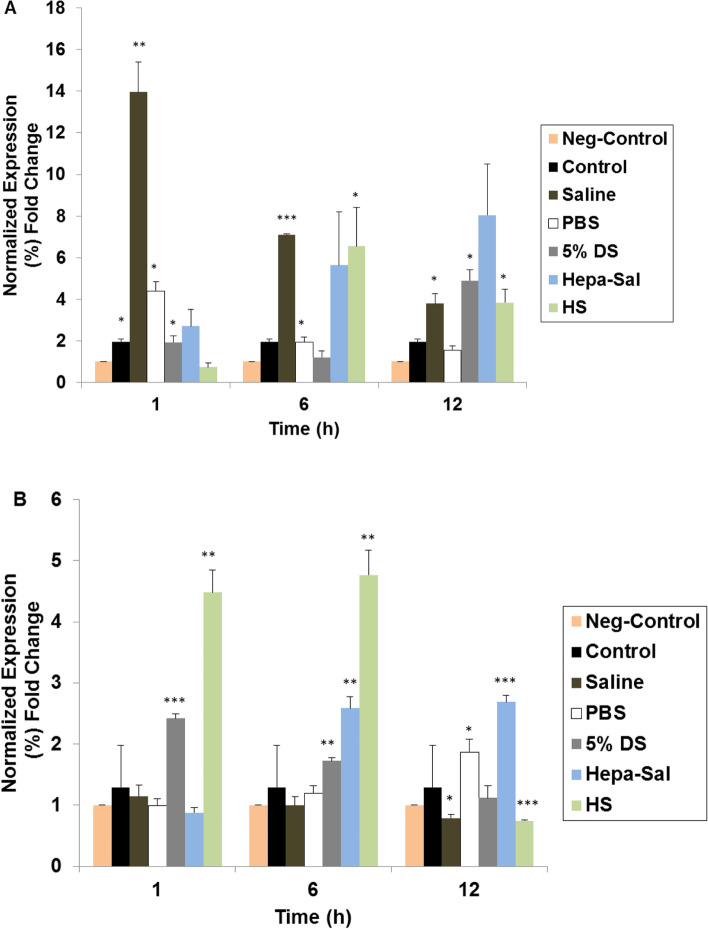
Post-storage differentiation ability of cADMSCs. cADMSCs were suspended in various carrier solutions at 4 °C for different time points. GAPDH was used as the housekeeping control gene. All mRNA data were normalized to the level of GAPDH and relative fold changes in expression level were measured. (**A**) The expression of COL2A was significantly higher in saline until 6 h and in Hepa-Sal at 12 h. PBS showed the lowest expression at 12 h of storage. (**B**) The expression of Sox9 of cADMSCs stored in HS was significantly higher at 1 and 6 h of storage. cADMSCs stored in Hepa-Sal at 12 h showed the highest expression of Sox9. Differentiated cells not stored in carrier solutions served as positive controls. The results are representative of three independent experiments. The bar graph represents the mean ± SD. cADMSCs - canine adipose-derived mesenchymal stem cells, GAPDH - Glyceraldehyde-3-Phosphate Dehydrogenase, COL2A - *collagen type IIa,* Saline - 0.9% saline, PBS - Phosphate-buffered saline, 5% DS - 5% Dextrose solution, Hepa-Sal - Heparin in saline, HS - Hartmann’s solution, NC - Negative control, SD - Standard deviation. (*P < 0.05), (**P < 0.001), (***P < 0.0001)

Quantitative reverse transcription-polymerase chain reaction (RT-qPCR) was performed to evaluate the expression of each target gene in the chondrogenic differentiation process. As shown in Fig. 5A and B, the chondrogenic differentiation potential of cADMSCs was affected over time in the different solutions compared with that of freshly harvested undifferentiated cADMSCs.

Compared to the other carrier solutions, the expression of *collagen type IIa* (COL2A) was significantly higher in cADMSCs stored in saline for 1 h and decreased over time, whereas Hepa-Sal showed opposite trends of increase in its expression from 1 h to 12 h (Fig. 5A). The lowest expression of COL2A was observed in PBS at 12 h. In Contrast, storage in HS at 1 and 6 h also showed higher expression of Sox9; however, it decreased drastically at 12 h, as it may have been affected by a longer period of storage (Fig. 5B). Hepa-Sal revealed the highest Sox9 expression, whereas its expression in cADMSCs stored in saline and 5% DS decreased over time (until 12 h).

## Discussion

Stem cells are a useful tool in the field of regenerative medicine, and promising stem cell-based cellular strategies are actively being developed to treat degenerative and fatal diseases [39]. In clinical settings, cell type is a significant criterion for stem cell therapy; other factors to consider include cell dose, route of administration, and most importantly, storage time [40, 41]. For transplantation, ADMSCs must be detached from the cell culture expansion vessel and immediately preserved in a carrier solution before administration to the patient. In practice, the prompt transplantation of ADMSCs is not always feasible under clinical conditions. Many cell transplantations and surgical processes, as well as long-distance transportation of ADMSCs, may increase the waiting time before transfusion. Currently, traditional storage methods including cryopreservation cause cell destruction due to the use of cryoprotectants such as dimethyl sulfoxide, and they have been replaced by supercooling [42], vitrification [43], high-subzero preservation [44], and various other novel methods for the long-term (months to years) storage of MSCs.

Considering that a pH that differs from the physiological pH can cause an unfavorable impact on cell viability, we measured the pH of the carrier solutions and found that all the solutions maintained an acceptable pH for parenteral transplantation.

In clinical application, an extensive increase of MSCs in vitro is required to attain adequate cell numbers. Serigano et al. reported the importance of many factors including cell confluence as prerequisites in optimizing the expansion of BM-MSCs [10], which may affect the biological properties of BM-MSCs. In our evaluation, cADMSCs stored in 5% DS showed a constant level of highest confluence until 12 h. Contrastingly, cADMSCs stored in PBS for 1, 6, and 12 h showed the lowest confluence at each time point compared with the other solutions. Overall, all the storage solutions showed better confluency compared to control conditions, which reveals that they may have some specific mechanism that can improve the confluency of MSCs. However, the precise mechanism by which these solutions improve cell proliferation during storage is unclear and should be investigated in future studies.

In our study, the viability of cADMSCs decreased rapidly over a short duration of storage. Our results showed > 72% viability in all solutions except for Hepa-Sal (63%) at 6 h of storage. Similarly, Sohn et al. found that after 6 h, the viability of human BMMSCs (hBMMSCs) at 4 °C or room temperature (RT) was > 85% [38]. Moreover, Pal et al. found the viability of hBMMSCs in saline, 5% DS, and Dulbecco’s PBS for up to 6 h of storage at 4 °C was > 90%, which was slightly greater than the viability observed in our study [45]. Additionally, Veronesi et al. found that the viability of BMMSCs stored in physiologic saline for 18 h was 83% [46], whereas Ra et al. observed that the average viability of hADMSCs was 85.4% after 72 h of storage [47]. Yan Chen et al. showed that the storage of human umbilical cord blood-derived MSCs (hUCBMSCs) in normal saline for 6 h at 4 °C and RT exhibited maximum viability of 83 and 77%, respectively [48]. Stanislaus et al. [49] compared the efficacy of physiologic saline, PBS, and Dulbecco’s Modified Eagle Medium (DMEM)-high glucose as temporary storage solutions for MSCs. They found that hUCBMSCs showed > 70% viability after storage in physiological saline for 72 h. Elisa et al. [50] compared saline and DMEM as storage solutions for hADMSCs and reported that both storage solutions resulted in > 70% viability after 48 h. Their conclusion is following our result that cADMSCs stored in saline for up to 12 h showed the best viability. Regarding PBS, we found that cADMSCs stored in PBS for 12 h at 4 °C showed approximately 65% viability, whereas Muraki et al. [51] found that the viability of hBMMSCs stored in PBS at 4 °C for 24 h was 81%. Besides, Stanislaus et al. [49] also reported the viability of hUCBMSCs stored in PBS for 24 h to be > 70%.

In this study, the viability of cADMSCs stored in 5% DS decreased from 1 h and remained > 61% at 12 h, which is lower than the corresponding values in the study by Chen et al. [48] who reported the viability of hUCBMSCs stored in 5% DS for 6 h to be 80%. Moreover, Patricia et al. found that at 4 °C, the viability of hADMSCs stored in 5% DS and HS for 48 h was maintained at approximately 80% [52]. However, we observed > 74% viability when cADMSCs were stored in HS for 12 h at 4 °C.

Similar to normal saline, cADMSCs stored in Hepa-Sal at various time points showed a gradual reduction in cell viability. Regardless, cADMSCs maintained > 61% viability until 12 h of storage. We were unable to compare our results with those of other studies [53–55] because despite determining the effects of administration of MSCs suspended in the various storage solutions, their viability before transplantation was not recorded.

A key property of MSCs is their high proliferation rate [48]. In our study, we found that the ability of cADMSCs to proliferate steadily decreased gradually. Storage in saline and 5% DS revealed similar rates of MSC proliferation until 6 h, whereas storage in PBS revealed the highest proliferation of cADMSCs. The rates of proliferation continued to decrease significantly until 12 h of storage, and cADMSCs stored in 5% DS had a significantly higher proliferation rate compared to the other carrier solutions. Our findings conform with those of Pal et al. [45] that storing MSCs for < 8 h at 4 °C in 5% DS and up to 6 h at 4 °C in saline constituted permissible limits for clinical transplantation. In Contrast, Bronzini et al. [56] reported that cADMSC-treated cells showed less sensitivity to apoptotic stimuli than freshly isolated cells when maintained for 12 h in the PBS at RT.

Self-renewal is a common feature of stem cells. Thus, we investigated the effect of carrier solutions on the self-renewal ability of cADMSCs at different time points. Consistent with the results of Sohn et al. [38] that the CFU ability of hBMMSCs decreased by approximately 50% after 2 h of storage in saline, we found that cADMSCs formed approximately 40 colonies or more until 6 h in saline, PBS, Hepa-Sal, and HS. Additionally, cADMSCs in all the solutions continued to form more than 38 colonies at 12 h of storage.

To determine whether pluripotency and surface markers of cADMSCs were affected by the carrier solutions, RT-PCR was performed, and the results were compared with untreated cells. At 1 h of storage, cADMSCs in 5% DS showed the highest expression of Nanog whereas cells in saline, PBS, Hepa-Sal, and HS showed an approximately similar expression of Nanog at 1 h. However, the expression level of Nanog at 12 h was significantly lower in all the solutions except saline. Sox2 is a pluripotency and self-renewal marker naturally expressed in MSCs at low levels in early passages and decreases with increasing passages [57]. The cADMSCs stored in saline and 5% DS for 1 h had the highest expression of Sox2. Similarly, Madonna et al. and Sujiwan et al. showed that a high concentration of dextrose upregulates the expression of Sox2 [58, 59]. Furthermore, cells stored in PBS for 12 h showed the lowest expression of Sox2 compared to that in cells stored in other solutions. The expression of positive MSC markers, CD90 and CD105, were also affected by carrier solutions. After 1 h of storage in Hepa-Sal, saline, and 5% DS, cADMSCs showed the highest expression of CD90 and had a moderate reduction in expression until 12 h, maintaining the highest expression in HS. Pal et al. found that MSCs expressed 99.5% positive CD90 when stored in 5% DS for 2 h [45]. Our results are similar to those of Ferda et al. who showed that human umbilical cord multipotent stromal cells showed a higher expression of CD90 until 36 h of storage in HS supplemented with 1% human serum albumin than in PBS [60]. Contrastingly, CD105 expression was higher in PBS than in any other solution at 12 h of storage.

To determine whether the biological function of cADMSCs was affected by the carrier solutions, the post-storage chondrogenic differentiation ability of cADMSCs in a time-dependent manner was investigated. Saline and HS showed a higher expression of COL2A and Sox9, respectively, at 6 h. The significant decrease of Sox9 expression in HS at 12 h can be a result of a longer duration of storage. cADMSCs stored in Hepa-Sal for 12 h showed the highest expression of both genes compared to other solutions. The higher chondrogenic ability of Hepa-Sal might be associated with the inherent nature of heparin as it ensures the secretion of endogenous growth factors such as transforming growth factor β1, vascular endothelial growth factor, and fibroblast growth factor from the cells, thereby promoting chondrogenesis [61, 62]. Besides, several studies have shown that the incorporation of heparin in hydrogel can improve the re-differentiation of differentiated chondrocytes and chondrocyte phenotypes [63, 64]. Chen et al. reported that when hUCBMSCs were stored in 0.9% saline, 5% DS, and other parenteral solutions for 6 h, MSCs from all solutions exhibited multi-differentiation (osteogenesis and adipogenesis) ability [48]. To date, there is no report conducted on the chondrogenic ability of MSCs on different storage solutions which remained unable to compare the results. Therefore, this subject needs to be uncovered in further study.

Using PBS or any other culture medium, such as DMEM, as a carrier solution is not approved by the FDA as they are suitable for in vitro use, not for transplantation [48]. There is evidence that the freshness and quality of hBMMSCs decrease after storage for 6 and 12 h in PBS [65]. Additionally, Boon et al. reported that a significant aggregation of hBMMSCs into cellular clumps when stored in PBS for 90 and 180 min at 0 °C may create an embolic hazard if delivered into blood vessels in cardiac applications and that it can block injection or infusion catheters applied for cell delivery during surgical operations, possibly affecting the overall effectiveness of transplantation therapy [66].

Exosomes are considered for the key paracrine factors that mediate the therapeutic activities of MSCs [67]. Ghadrdoost et al. transplanted MSCs combined with exogenous heparin in the treatment of cardiac infarction in rabbits; the efficacy of this treatment could be attributed to the heparin-mediated enhanced paracrine activity of MSCs [68]. Particularly, the exogenous heparin promoted the expression level of growth factors such as vascular endothelial growth factor. Accordingly, future studies should test the impact of our test carrier solutions on the yield and quality of MSC-derived exosomes for disease therapy. Furthermore, there is recent evidence of the protective use of various other solutions for the storage of MSCs such as University of Wisconsin (UW solution), Hank’s balanced salt solution (HBSS), and histidine-tryptophan-ketoglutarate (HTK) solution [69, 70]. A future comparative study of our selected short-term storage solutions with the hypothermic preservative solution (UW solution, HBSS, or HTK formulation) commonly implemented in solid organ transportation [71] will be appropriate for the study aims and clinical approaches.. However, UW solution and HTK solution are not appropriate to inject directly in the body whereas our selected carrier solutions can be applied for the cell transplantation what makes them a superior storage solution for having higher cell viability, reduced cost, and faster procedure.

Our results suggest that ADMSCs should be used within 6 h or not more than 12 h to ensure a high survival rate of cells and the efficacy of therapy. Moreover, an appropriate carrier solution should be selected based on the patient’s preexisting condition. For example, 5% DS should be avoided in patients with diabetes or hyperglycemia, and Hepa-Sal could be prioritized for cartilage regeneration and avoided in cases involving frequent bleeding tendency. Saline and HS can also be utilized as carrier solutions of ADMSCs depending on the necessity of the therapy. Notably, our study suggests that non-FDA-approved PBS should be replaced, preferably with Hepa-Sal to reduce the risk of embolisms, or any other FDA-approved solutions to increase cell viability. Further investigation on the effect of PBS on MSCs is warranted. Moreover, our findings need to be verified using in vivo models, and osteogenesis should also be examined.

## Conclusion

The viability and efficacy of cADMSCs decreased over time when stored in different carrier solutions. Optimal storage is a requirement for the maximum utilization of their potential in clinical application. When storage is mandatory, ADMSCs can be stored in carrier solutions such as saline, 5% DS, Hepa-Sal, or HS at 4 °C, preferably for less than 6 h and no longer than 12 h. We also recommend replacing PBS with Hepa-Sal or any other FDA-approved carrier solution to ensure high cell viability, proliferation rates, differentiation ability, and native gene expression levels.

## Methods

### Isolation, culture, and expansion of ADMSCs

cADMSCs were isolated from the gluteal subcutaneous fat of a two-year-old Beagle that was primarily used for another objective in surgery research (Institutional Animal Care and Use approval number, KU18169–1). All the procedures were conducted according to the animal care and ethical guidelines of Konkuk University and Institutional Animal Care and Use guidelines for animal research. A previously described modified method was used to isolate the cells [72]. Briefly, the fat tissue was collected using the surgical procedure and washed thoroughly with PBS (Biowest, Seoul, South Korea). Then, the tissue was weighed (12 g) and digested with 0.1% collagenase type I (Worthington Biochemical Co., New Jersey, USA) prepared in sterile PBS and 1% penicillin-streptomycin (Gibco, New York, USA) for 1 h at 37 °C in a thermostatic water bath oscillator (Taitec Corporation, Saitama, Japan). The homogenate was centrifuged at 300×*g* for 10 min to distinguish the floating adipocytes from the stromal vascular fraction. Then, the supernatant was carefully discarded and the stromal vascular fraction was resuspended in DMEM-F12: (nutrient mixture) (Gibco), filtered with a 70-μm cell strainer (SPL Life Sciences, Gyeonggi-do, South Korea), and centrifuged at 150×*g* for 5 min to acquire a high-density cell pellet. The cells were plated at a density of 5 × 10^4^ cells/cm^2^ in a T175 cm^2^ flask (SPL Life Sciences) and expanded in DMEM-F12supplemented with 10% fetal bovine serum (WELGENE Inc., Gyeongsangbuk-do, South Korea) and 1% penicillin-streptomycin in a humidified environment with 5% CO_2_ at 37 °C. The medium was replenished until 72 h, followed by a complete medium change on day 5 with the continuous microscopic observation of cell condition and attachment. We passaged the cells after reaching 80% confluence, cells were harvested and counted. Next, we suspended 5 × 10^5^ -5 × 10^6^ cADMSCs/vial (*n* = 10) containing 1 mL cell banker (Cell Banker 1, Nippon Zenyaku Kogyo Co., Ltd., Fukushima, Japan) at passage 1 for cryopreservation [73]. Cells from passage 2 were used for further studies [74].

### Cell morphology and proliferation

Cell morphology was investigated via monolayer culture as described previously [75]. Briefly, cryopreserved cells were thawed in a water bath at 37 °C and cultured in 6-well plates (SPL Life Sciences) at 2 × 10^4^ cells/cm^2^. When cells reached 80–90% confluency, they were trypsinized by 0.25% trypsin (Gibco) and stored in tested carrier solutions, namely saline (Greenflex, Gyeonggi-do, South Korea), PBS, 5% DS (Greenflex), Hepa-Sal (JW Pharmaceutical, Seoul, South Korea), and HS (Safe-Flex Health Care, Seoul, South Korea) for 1, 6, and 12 h at 4 °C (Table 2). After the end of the storage period, the cells were re-seeded into the complete growth medium in a humidified environment with 5% CO_2_ at 37 °C, and the post-storage morphology and confluence of the attached cells at 24 h were observed using an inverted photomicroscope (Olympus Korea Co. Ltd., Seoul, South Korea). The obtained pictures were analyzed using ImageJ software (National Institutes of Health, USA). Untreated cells were used as controls. All experiments were performed in triplicates.

**Table 2:** Components of the selected carrier solutions

Solution	Components	Tonicity	Stated pH	Osmolarity	U.S. FDA-Approval
0.9% Saline	9 g/L Sodium chloride	Isotonic	5.0 (4.5–7)	308	Yes
PBS	Sodium chloride, potassium chloride, disodium phosphate, potassium phosphate	Isotonic	7.4	280–315	No
5% DS	Dextrose, hydrous 5 g in water	Hypotonic	4.3 (3.2–6.5)	278	Yes
Hepa-Sal	Sodium chloride, diphasic sodium phosphate, citric acid anhydrous, heptahydrate, water for injection	Isotonic	6.8–7.2	360	Yes
Hartmann solution	Sodium chloride, sodium lactate, potassium chloride and calcium chloride	Hypotonic	5–7	279	Yes

*Saline* 0.9% saline, *PBS* Phosphate-buffered saline, *5% DS* 5% Dextrose solution, *Hepa-Sal* Heparin in saline, *HS* Hartmann’s solution

### pH measurements

In this experiment, we aimed to measure the pH range of the tested carrier solutions after the storage of cADMSCs in a time-dependent manner. cADMSCs were dissociated by 0.25% trypsin and were washed twice with PBS followed by resuspension at a concentration of 1 × 10^6^ cells/mL in selected carrier solutions for 1, 6, and 12 h at 4 °C. After storage, the supernatant was collected and pH was measured in triplicates using a pH meter (Mettler Toledo, Giessen, Germany). Fresh solutions without cells at time 0 were used as controls.

### Proliferation assay

To assess the proliferation capacity of post-storage cADMSCs, a cell proliferation assay was conducted using CCK-8 (Dojindo Molecular Technologies, Maryland, USA) following the manufacturer’s instructions. Briefly, post-storage, 5 × 10^3^ cells per well were seeded in 96-well plates (SPL Life Sciences) until they reached confluency. We set untreated cells as controls. The culture media was removed and 100 μL of fresh media containing 10 μL of CCK-8 was added to each well. The plate was then incubated for 3 h at 37 °C and 5% CO_2,_ and the absorbance was measured at 450 nm using a spectrophotometer (SpectraMax, Molecular Devices, CA, USA). All experiments were performed in triplicates.

### MTT assay

The viability of post storage cADMSCs was measured using the MTT (3,4,5 – dimethylthiazol-2-yl)-2–5-diphenyltetrazolium bromide) assay kit (Abcam cat. ab211019, Gyeonggi-do, South Korea) following the manufacturer’s protocol. Briefly, post-storage, 10 × 10^3^ cells/well were seeded in 96-well plates (SPL Life Sciences) until they reached confluency. Untreated cells were used as controls in the interpretation of the data. Cells were incubated with the MTT solution for 3 h at 37 °C and 5% CO_2,_ followed by the addition of MTT solvent for 15 min. Optical density was measured at 590 nm using a spectrophotometer. All experiments were performed in triplicates.

### Colony-forming unit assay

We measured the CFU of cADMSCs at different time points by seeding 200 cells/100 mm dish (SPL Life Sciences) post-storage in triplicates according to the previously described method [72]. Cells were stained at day 14 with 1% crystal violet (Sigma-Aldrich, Missouri, USA) in methanol (Sigma-Aldrich), washed, and photographed. Then colonies, with ≥50 cells were counted manually.

### Chondrogenic differentiation

For chondrogenic differentiation, 1 × 10^6^ cells were cultured in 6-well plates (SPL Life Sciences) [76]. After reaching 60–80% confluency, the pellets were made by transferring cells into a 15-mL tube (SPL Life Sciences) followed by centrifugation at 1600 rpm for 5 min. The supernatant was discarded and replaced with 1 mL of chondrogenic differentiation medium (Cell Applications, Inc., CA, USA). The media were changed every alternative day for 14 days.

### Gene expression analysis

After storage in carrier solutions, RT-PCR was performed as described previously [72]. The total RNA was extracted by TRI reagent® (Favorgen Biotech Corp., Kaohsiung, Taiwan) application according to the manufacturer’s protocol. The RNA concentration was measured using NanoDrop software (Thermo Fischer Scientific, Seoul, South Korea). Using 2 μg of the total RNA, the complementary deoxyribonucleic acid (cDNA) was synthesized using the 1st Strand cDNA Synthesis Kit (Takara, Shiga, Japan) following the manufacturer’s guidelines. Gene expression of the canine housekeeping gene glyceraldehyde 3-phosphate dehydrogenase (GAPDH) (Bioneer Corporation, Daejeon, Korea) [77], Nanog, Sox2 [72] and a cluster of differentiation molecules such as CD45, CD90, and CD105 (the most commonly found negative and positive markers respectively for cADMSCs [78]) were investigated. To prepare 20 μL of the total reaction volume, 17 μL of distilled water, 1 μL of cDNA, and 1 μL each of 10 pmol of reverse and forward primers were used. PCR amplification was performed with 35 cycles at 60 °C for 30 s using the GeneAmp® PCR system 9700 (Applied BioSystems, Foster City, CA, USA). The PCR products were marked by gel electrophoresis on 2% agarose (Duchefa Biochemie, Haarlem, Netherland) using a DNA ladder (Thermo Fischer Scientific). Images were digitally detected and recorded using Gel Doc (Sigma-Aldrich).

For the determination of chondrogenesis, the total RNA was isolated on day 14 from undifferentiated and differentiated cells and cDNA was synthesized. To determine the expression of Sox9 and COL2A, RT-qPCR was conducted using Power SYBR Green reagents in a 7500 RT-PCR System (Applied Biosystems, California, USA). Gene expression levels were calculated using the 2^(−ΔCt)^ method relative to GAPDH as a reference gene [79]. The PCR primer sequences for differentiation are summarized in Table 3.

**Table 3:** List of PCR primer sequences

Marker	Gene	Primer sequence (5′–3′)	Amplification Size (bp)
Housekeeping	GAPDH	F - GGAATCCACTGGCGTCTTCA R - GGTTCACGCCCATCACAAAC	122
Stemness	Nanog	F - GAATAACCCGAATTGGAGCAG R - AGCGATTCCTCTTCACAGTTG	141
	Sox2	F - AACCCCAAGATGCACAACTC R - CGGGGCCGGTATTTATAATC	152
MSC markers	CD45	F - CTCACGCACACAGGCTCGCA R - CCCACCCACTGGCACTGCTG	159
	CD90	F - CAGAACACCTCATGGCTGCTGT R - GGAGAAACCAGACAGAAGCGA	329
	CD105	F - GGTTCACTGCATCAACATGG R - AAGCTGAAGCGCACATCACC	278
	SOX9	F - GCTCGCAGTACGACTACACT R - GTTCATGTAGGTGAAGGTGG	101
	COL2A	F - GAAACTCTGCCACCCTGAAT R- GCTCCACCAGTTCTTCTTGG	145

*F* Forward, *R* Reverse, *FDA* U.S. Food and Drug Administration (FDA), *bp* base pair

### Statistical analysis

The results were expressed as the mean ± standard deviation and analyzed by GraphPad Prism 6.0 (GraphPad Software, La Jolla, CA, USA) software, and the differences in mean number were determined by a student’s t-test. A probability level of *P < 0.05, ** *P* < 0.001, and ***P < 0.0001 was considered statistically significant.

## Data Availability

The datasets used and/or analyzed during the current study are available from the corresponding author on reasonable request.

## Abbreviations

ADMSCs: Adipose-derived mesenchymal stem cells
BM: Bone marrow
BMMSCs: Bone marrow-derived mesenchymal stem cells
cADMSCs: Canine ADMSCs
CCK-8: Cell Counting Kit-8
CD: Cluster of differentiation
cDNA: Complementary deoxyribonucleic acid
CFU: Colony-forming unit
dH_2_O: Deionized water
5% DS: 5% Dextrose solution
DMEM-F12: Dulbecco’s modified Eagle’s Medium: Nutrient Mixture
F: Forward
FDA: Food and Drug Administration
GAPDH: Glyceraldehyde 3- phosphate dehydrogenase
Hepa-Sal: Heparin in saline
HS: Hartmann’s solution
HBSS: Hank’s balanced salt solution
HTK: Histidine-tryptophan-ketoglutarate
hADMSCs: Human ADMSCs
hBMMSCs: Human BMMSCs
hUCBMSCs: Human umbilical cord blood-derived mesenchymal stem cells
MSCs: Mesenchymal stem cells
MTT: (3,4,5 – dimethylthiazol-2-yl)-2–5-diphenyltetrazolium bromide)
PBS: Phosphate-buffered saline
RT: Room temperature
RT-PCR: Reverse transcription-polymerase chain reaction
R: Reverse
Saline: 0.9% saline
UW solution: University of Wisconsin solution
